# Augmented Reality in Ward Round-Based Simulation: Exploring Student Experiences and Impact on Confidence

**DOI:** 10.7759/cureus.73847

**Published:** 2024-11-17

**Authors:** Christopher Rainforth, Aneeta John, Alison Brown

**Affiliations:** 1 Medicine, Great Western Hospitals NHS Foundation Trust, Swindon, GBR

**Keywords:** augmented reality, medical education, medical education technology, medical simulation, ward round-based simulation

## Abstract

Introduction: Doctors in training make substantial contributions to ward rounds. Despite this, formal education in this area is lacking, leading to diminished confidence and competence among undergraduate students and trainees. Ward round-based simulation (WRBS) has shown promise in improving clinical and decision-making skills. This quasi-experimental mixed-methods study aims to explore student experiences of augmented reality (AR) in simulated medical ward rounds. Specifically, it seeks to compare AR to existing traditional simulation modalities (TSM), manikin, and actor, to evaluate its viability as an alternative and to explore the impact of a mixed-modality simulated ward round on student confidence levels in performing ward round tasks.

Materials and Methods: Fourteen participants engaged in a simulated ward round involving three cases: an actor as a patient, a manikin, and an AR case. Guided by pre- and post-simulation questionnaires, confidence levels around WRBS were explored using Wilcoxon's signed-rank test. Additionally, thematic analysis of semi-structured focus groups explored the use of AR in simulation compared to TSM.

Results: Significant improvements in confidence shown through the pre- and post-simulation questionnaires (p < 0.019) were found using Wilcoxon’s signed-rank test across all 11 questions, with effect sizes (r) ranging from 0.65 to 0.9. The highest effect size observed (r = 0.9) asked about the change in confidence when starting or stopping medications during a ward round (p = 0.002). Qualitative analysis of focus group discussions identified key themes such as the visual accuracy of AR, the need for interactivity, and the cognitive load of mixed-method simulations.

Conclusion: The findings suggest that AR can significantly enhance medical training by providing realistic and immersive learning experiences, although further refinements are necessary to improve interactivity and reduce cognitive demands. This study highlights the potential for integrating innovative technologies to better prepare students for clinical practise and provides further insight into the practicalities of using AR in medical education.

## Introduction

Medical schools work hard to create robust curricula that generate competent and safe junior doctors equipped with the necessary knowledge and skills. Despite this, medical students often feel unprepared for the transition into working as a junior doctor [1]. Specific concerns include managing emergency situations and a lack of familiarity with hospital procedures. Taking part in a ward round within the multidisciplinary team (MDT) is an important skill for junior doctors. Ward rounds allow the MDT within a hospital setting to assess patients and formulate care plans throughout a patient's admission and importantly provide an opportunity to communicate these plans to the patient [2]. They usually happen daily and involve senior clinicians, junior doctors, nurses, physiotherapists, and occupational therapists to co-ordinate patient care. The Royal College of Physicians and Royal College of Nurses have set out guidance on the ‘modern ward round’ and recommendations on how they are best executed [3]. The key messages they advise include that it is ‘well led’ and ‘structured’, uses ‘effective teams’, has ‘patient involvement’, and includes ‘education, learning, and improvement’ [3,4]. Within this, junior doctors play a key role in documenting discussions and examination findings, reviewing previous investigations, and informing the team about any communications with other specialties [5]. The ward round presents a unique opportunity to learn by observation; it is a source of informal and formal teaching [6]. However, formal training for medical students in how to perform these tasks and participate appropriately is lacking leading to a deficit in skillset [5]. Literature suggests that simulation and feedback can be used to equip students to manage common ward-based tasks and distractions [7].

During clinical years, medical students spend most of their time on the wards engaging in tasks that arise from the ward rounds under supervision from the junior doctors. Medical students' roles within a ward round are not well defined; this can lead to unfavourable attitudes, which act as barriers to learning [8]. It has been shown that ward round-based simulation (WRBS) does improve clinical and decision-making skills [9,10]. Simulation is a useful modality to supplement training in real-life scenarios like ward rounds, providing a safe platform for students to make mistakes and reflect without compromising their wellbeing or professional identity [11]. Simulation approaches and modalities have been heavily debated within the literature [12]. Discussion centres around fidelity, the type of skills taught, and the approach to feedback. The aim of simulation is to have a degree of fidelity, which allows for student engagement and maintains a level of realism to facilitate desirable clinical performance with effective participation to achieve learning outcomes. Realistic characteristics and certain clinical signs are difficult to replicate in manikins such as changes to skin colour and facial expressions [13]. Additionally, response to intervention is limited and may not match the learners' expectations. Advances in technology allow augmented reality (AR) to be used as a platform for improving fidelity during simulation scenarios [14,15]. AR is a technology that adds virtual content to the physical real world with the potential to offer highly realistic and accurate simulations as it augments the perception of reality. Existing literature on the use of AR in medical training predominantly focuses on post-graduate education, where it is applied to specific surgical or interventional procedures. In contrast, studies involving AR at the undergraduate level primarily concentrate on teaching anatomy, with limited applications in other contexts [16-18]. This study recognised the need for increased ward round teaching and combined the already effective WRBS with a novel AR element to the simulation. The impact of AR and the importance of fidelity, within a simulation, particularly a ward round-based scenario, has been explored.

## Materials and methods

Ethical approval

This study was reviewed and approved by the Swindon Academy Medical Education Research Committee. The committee determined that the project met the minimum ethical standards for conducting medical education research. Approval for the study was granted on 09/02/24, and the reference code AB0224 was assigned to this project. All participants provided informed consent prior to their involvement in the study, in compliance with ethical guidelines. Students were recruited to participate in the study via email, and 14 students from three different universities and across two different year groups enrolled. Each volunteer student signed a consent form to state they were happy to participate.

Study set-up and structure

This study utilised a quasi-experimental mixed-methods design, incorporating both qualitative (focus groups) and quantitative (pre- and post-simulation questionnaires) approaches to evaluate the impact of AR-based simulation on student confidence during ward rounds. Five WRBS sessions on five separate occasions were carried out. Each session took 90 minutes with 45 minutes in the simulation itself and 45 minutes for the pre-brief, debrief, and focus groups. The ward was made up of three patients (Appendix 1) with three different modalities used to portray the patients: AR, a manikin, and an actor. The same three faculty members facilitated the simulation and debrief, assuming the same roles each time: nurse, senior clinician, and actor for the third patient. The WRBS took place in a large room with examination couches as patient beds. The patients were kept in the same location of the room each time the scenario ran. The equipment used to generate the AR patient included one Microsoft HoloLens 2 device (Microsoft Corp., Redmond, WA, US); the GigXR software with HoloPatient Module provided a loop recording of the AR patient [19]. This was visible to the students through either the headset or mobile phones with access to the AR application. In the study, one student volunteer wore a headset, while the other students in the group viewed the same visuals through two mobile phone screens. The students assessed and independently managed each patient as they would in a ward round, prioritising and delegating jobs as they deemed appropriate. The scenario ended when all three patients had a management plan. The debrief for the clinical aspect of the WRBS followed straight away after the scenario finished and lasted about 15 to 20 minutes. This was followed by the focus group interview for the remainder of the session, which was recorded using Microsoft Teams (Microsoft Corp., Redmond, WA, US).

Five semi-structured focus groups were generated: four groups of three students and one with two due to an unplanned absence. Each focus group of students was asked the same three questions (Appendix 2) to spark a discussion about their experience of using AR. If needed, follow-up questions were asked to clarify any points raised. All groups were also given the opportunity to make any general comments. A thematic analysis approach was employed to identify and report common themes present in the five interview transcripts. Steps taken included familiarisation with the data, generating initial codes, searching for themes, reviewing themes, defining and naming themes, and producing a report [20]. The recordings of each interview were transcribed, and the transcripts were then corroborated with the audio recordings to ensure accuracy, with any errors being corrected during this process. Multiple reviews of the transcripts facilitated familiarisation with the data. Initial codes were generated manually with an Excel sheet (Microsoft Corp., Redmond, WA, US) used to log codes and corresponding quotes. From these codes, major and minor themes emerged. Major themes were identified based on codes shared across all five focus groups, indicating common experiences and perceptions. Minor themes, while frequently discussed, represented experiences specific to individual groups rather than the entire sample.

Pre- and post-simulation questionnaires were completed (Appendix 3) where each student rated their confidence in different aspects of the ward round. The questionnaire was developed and discussed with a group of clinical teaching fellows experienced in delivering simulations. This collaboration was essential in ratifying the questions and ensuring their relevance and appropriateness for the study. However, no formal validation process was conducted. Future studies may benefit from pilot testing or reliability assessments to further ensure the robustness of the tool. The questionnaires used a Likert scale response format to collect more accurate and reliable student responses. Wilcoxon’s signed-rank test was used to analyse this paired data by assessing changes in the participants’ confidence levels before and after the simulation. The test then determined if there was a statistically significant change in confidence reflected in the p-values. Given the non-normally distributed data and small sample size, Wilcoxon’s signed-rank test was chosen as it is specifically designed for paired samples and does not assume normality.

## Results

Quantitative results

Our study evaluated the efficacy of AR and traditional simulation modalities (TSM) in enhancing medical students' confidence during ward rounds using pre- and post-questionnaires and Wilcoxon's signed-rank test for analysis. The results demonstrated significant improvements in confidence across all nine questions. The results are summarised in Table 1.

**Table 1:** Wilcoxon's signed-rank test analysis of the pre- and post-simulation questionnaires, demonstrating effect sizes on self-reported confidence levels. SBAR: situation, background, assessment, and recommendation; Mdn: median

Comparison	Pre (Mdn)	Post (Mdn)	Z	p	Effect size (r)
How confident do you feel actively participating in a ward round led by someone more senior? For instance, scribing.	4	7	2.9	0.002	0.8
How confident do you feel in undertaking a ward round by yourself?	1.5	5	2.9	0.002	0.9
How confident do you feel in your assessment and examination of the patient during a ward round?	5	6	2.1	0.019	0.7
How confident do you feel coming up with the plan following your assessment of the patient during a ward round?	4	6	2.8	0.002	0.8
How confident do you feel requesting investigations following a ward round?	3	5.5	2.8	0.003	0.9
How confident do you feel about your SBAR handovers? For instance, when vetting a scan.	4.5	6	2.2	0.016	0.65
How confident do you feel knowing when and how to escalate problems during the ward round?	4	6	2.7	0.003	0.8
How confident do you feel stopping and/or starting medications during a ward round?	2	6	2.9	0.002	0.9
How confident do you feel about creating and prioritising a job list following a ward round?	4	6	2.7	0.004	0.8

**Figure 1:**
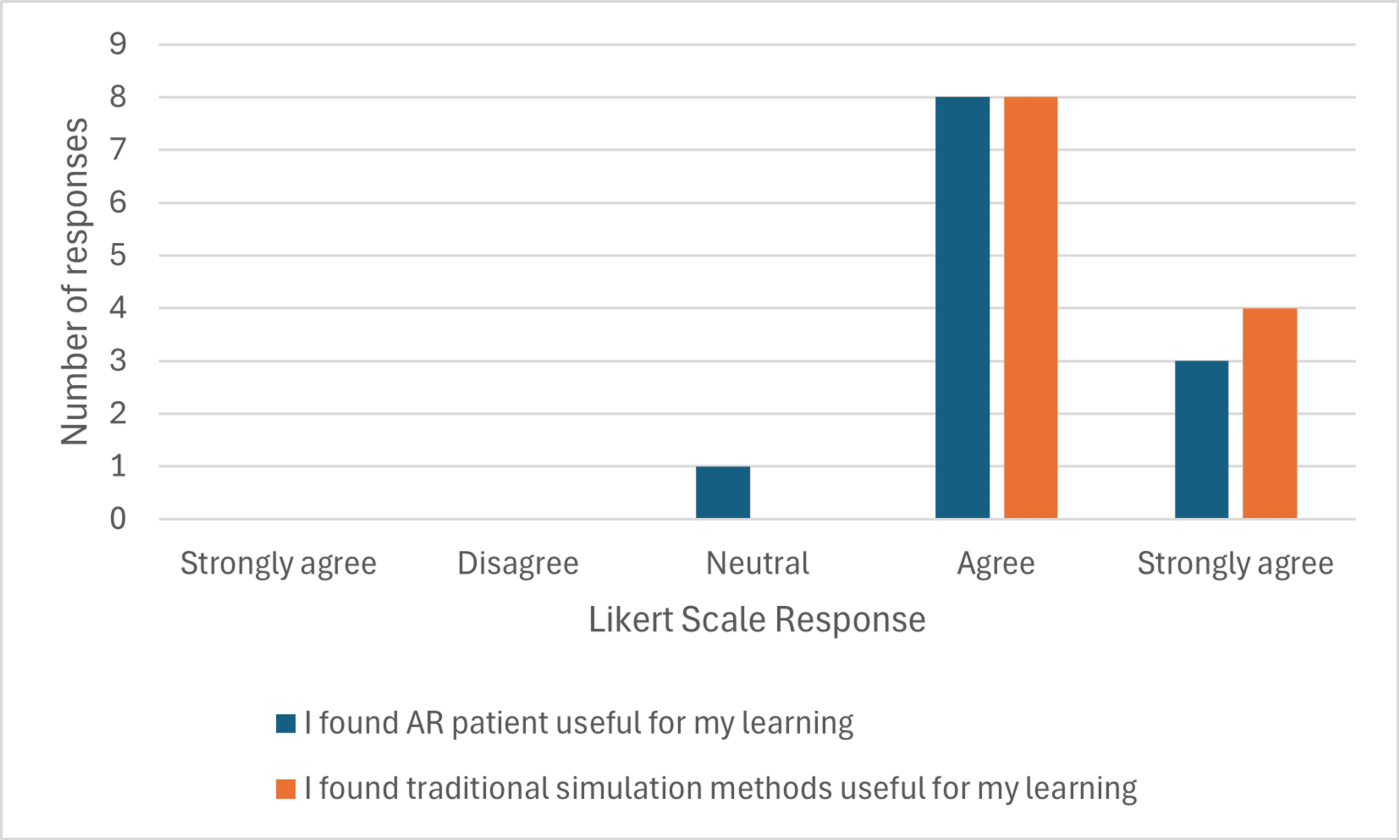
Student responses comparing AR against TSM regarding their usefulness as a learning tool. AR: augmented reality; TSM: traditional simulation modalities

Wilcoxon's signed-rank test demonstrates significant improvements in confidence across all tasks following the simulations, with p-values below 0.05 in all comparisons. Notably, question 8, which assessed confidence in stopping and/or starting medications, exhibited the highest effect size (r = 0.8809), indicating a substantial gain in confidence for this task. Two final questions were used in the pre- and post-questionnaires to compare the different modalities of simulation used, as demonstrated in Figure 1.

Qualitative results

The focus group discussion centres on the impact of AR and the relevance of ward round simulation. Thematic analysis revealed three major themes and three minor themes.

Major themes

Major themes (Table 2) were generated from codes that appeared in all five transcripts, reflecting that this is a widely shared observation.

**Table 2:** Table showing direct quotations, taken from student focus groups, to support each major theme. AR: augmented reality

Major theme	Quotation	Focus group
AR is visually accurate	'It was quite nice to see, like an unwell patient where you could actually pick up clinical signs that you extracted. And he looked unwell, whereas you can have an actor that will look well, they will try and everything, but they don't look like they are sick or a manikin'.	4
Each simulation modality had different strengths	'And I think for me, like the AR would be better for recognising signs, but yeah for communication an actor was definitely better'.	2
AR experience is restricted by the lack of interactions	'I don't think it actually changed my confidence, I think. A lot of maybe where we need to work on confidence or where you need to be confident individuals like actually speaking to people. And we confident enough your examination confidence with questions you're asking and with the AR you didn't get that out of this? That's just for me though'.	3

AR is visually accurate: The sub-themes identified are that it allows for independent judgement, allows for the recognition of sick patients, and provides realistic visual prompts. An extensive point of discussion was the visual impact of the AR patient. Many students reported the satisfaction of being able to recognise clinical signs themselves. The students identify that the visual realism of AR is a true strength of this teaching method when compared to other teaching methods. Visual realism allows students to inspect the patient from the end of the bed, as they would in a clinical scenario, to ascertain important clinical information. The AR patient helped students practise this inspection and identify that this is an unwell patient, just by looking at them from the end of the bed. This ability to make independent judgments significantly impacted students' confidence. Students valued this independence as it contrasts with TSM, where visual findings are not always replicable, or students must ask facilitators for verbal descriptions.

Each simulation modality has different strengths: The sub-themes identified are the order of fidelity, with actor ranked highest followed by AR and manikin; the acquisition of different useful skills from simulation; and mixed responses regarding confidence. Students discussed that each modality has its respective place in an educational setting and has its own strengths. Despite the positive remarks on the AR being visually accurate, overall, the actor provided the most realism. The element of communication and interaction with the actor patient was greatly valued. Interestingly, even though the students were able to communicate with the manikin, this was felt to be the worst in terms of fidelity. The mixed-methods approach meant that each patient had a slightly different focus-the AR patient relied on the student's ability to recognise clinical signs, the actor played a delirious patient focused on clinical prioritisation and examination skills, and the manikin had a greater focus on communicating with a patient wanting to be discharged. However, when asked to compare these methods to comment on the impact on their overall learning experience and confidence levels, there were mixed opinions; some students felt that learning was equivocal, and some students felt that AR had no impact on confidence levels overall.

AR experience is restricted by the lack of interactions: The sub-themes identified are that AR can be improved to incorporate more elements, communication is an important aspect, and interaction is crucial for building confidence. The AR technology used in this simulation displayed a loop recording of a patient. Students consistently identified the lack of interactivity as a significant restriction of AR technology in the simulation. Many felt that the inability to directly interact with the AR patient diminished the overall learning experience. While the AR patient was visually more representative of a sick patient than a manikin, the inability to engage in two-way communication or perform physical examinations was a major drawback. Students described that this even makes the patient ‘forgettable’ during the rest of the ward round simulation when their attention is elsewhere. Unlike with actor patients, students could not ask the AR patient questions or receive responses, limiting their ability to practise communication skills. This also creates an emotional barrier; the AR patient does not show any real-time feedback in behaviour reducing the need for students to display empathy and compassion. Students noted that they could not interact directly with the AR patient, which would have enhanced the realism and educational value of the simulation and thereby contributed more to their confidence levels. Hence, focus group discussions touched on suggestions to improve the current AR model, incorporate more interactive elements, and make it more comparable to the actor.

Minor themes

The themes identified as minor (Table 3) were less widely discussed than major themes but were nonetheless significant points in the discussions. Students’ feedback on logistical barriers, the novelty of AR, increased cognitive load from mixed-methods simulations, and their varied previous ward round experiences provide valuable insights into the potential and challenges of integrating AR into medical training.

**Table 3:** Table showing direct quotations, taken from student focus groups, to support each minor theme. AR: augmented reality

Minor theme	Quotation	Focus group
AR is a novel approach	'I mean, it was. It was fresh to see. I got quite excited by it. Something that I've never seen before and it kind of lived up to my hopes on the types of augmented reality'.	1
AR has logistical barriers	'Maybe disjointed isn’t the right word. I don’t know, what is the right word. Maybe it's a bit jumpy. And I think just everyone having a headset would obviously be better, but that’s obviously a resource thing'.	2
Mixed-methods simulation increases cognitive load	'And this is new technology. And so there is that kind of novelty to you know, you're arriving, you see the goggles and it it's we're used to seeing actors. We used to see manikins. If it felt not very integrated, not for any fault of the design of it, but just because of where we are at as understanding of technology, is not something we've had before. It's a novelty, so it sticks out a lot'.	3
Varied previous clinical ward round experiences	'You don't feel very confident or a useful part of the ward round you’re, kind of, just like the curtain closer. (Yeah.) So, like, it's not until, like you have SIMS where you're in an environment where you can practise making mistakes and not being embarrassed in front of a consultant'.	5

AR is a novel approach: Students highlighted the novelty of AR technology as a significant aspect of their experience. They described it as ‘fresh’ and ‘exciting’, noting that it introduced a new dimension to their learning. Many had never encountered AR in a medical context before, and its introduction lived up to their expectations. Some students expressed initial uncertainty about what to expect, given their traditional medical training. The novelty of AR stood out, making the experience memorable and enjoyable. The use of AR provided a unique and engaging way to visualise clinical scenarios that would be challenging to replicate with TSM. The potential for AR to transform learning by allowing students to see different patients and scenarios in their own space was particularly appealing.

AR has logistical barriers: Students identified several logistical challenges associated with using AR technology. The primary issue was inconsistent access to AR headsets, which led to confusion about whether all students were seeing the same observations. Some students had headsets, while others relied on mobile devices, causing disparities in the experience. This use of additional devices made it challenging to multitask, especially when documenting their ward round plan for the AR patient. A significant barrier was the lack of integration of AR with other simulation elements. This was compounded by the need to switch between AR and non-AR components during the simulation, which disrupted the flow and consistency of the exercise.

Mixed-methods simulation increases cognitive load: Despite its exciting potential, students acknowledged that the technology felt somewhat isolated due to their unfamiliarity with it. The mixed-methods approach, incorporating AR alongside TSM, significantly impacted cognitive load. The transition between different simulation modalities required students to adapt continuously, adding to their cognitive burden. The AR patient, only visible through a headset or device, required a conscious effort to remember, unlike the actor and manikin patients who were physically present in the room.

Varied previous clinical ward round experiences:* *Students reported a wide range of previous experiences with clinical ward rounds. Many expressed that medical students often do not have ample opportunities to observe acutely sick patients during their training. There was also a sense of fear of embarrassment from making mistakes or ‘actively hindering’ the process, which hindered active participation in clinical ward rounds.

## Discussion

Study limitations

One notable limitation of the study is the inherent subjectivity in measuring 'confidence' levels, which relies on self-reported data from participants. This introduces a risk of response bias, as participants might have felt compelled to provide more positive ratings to align with the perceived expectations of the researchers [21]. In order to mitigate this, anonymised responses were collected using the questionnaires. Another challenge in this study is the difficulty in directly comparing AR with TSM. Given that simulation, in general, is a proven and effective learning tool [12], it is reasonable to expect an increase in confidence regardless of the simulation modality used. Furthermore, the small sample size limits the generalisability of the findings, making it difficult to draw broader conclusions. Thus, while both AR and TSM showed significant gains in confidence, these factors highlight the complexities in interpreting the precise impact of each simulation type.

Quantitative insights

This WRBS study provides evidence that both AR and standard simulations significantly enhance confidence in performing ward round tasks, highlighting the potential value of incorporating innovative simulation technologies into medical training curricula [9,10,12]. Substantial improvements in confidence were observed across all tasks following the simulations; however, the relative contributions of AR compared to TSM remain unclear. Question 8, which examined the confidence in stopping and/or starting medications, had the highest effect size (r = 0.8809), indicating the most substantial gain in confidence. The AR case involved prescribing new medications, while TSM also encompassed the same. Consequently, it is difficult to conclude which modality had the largest impact on the students’ confidence with prescribing, or indeed whether simply completing a WRBS was the most important aspect in this regard.

A meta-analysis of AR in medical education has shown that AR is effective in improving participant confidence, with some studies indicating that AR yields greater effects under controlled conditions [18]. The findings of our study align with existing literature on the effectiveness of AR in improving confidence and skill acquisition in medical education settings [18,22]. The demonstrated effectiveness of AR in boosting confidence suggests that incorporating AR into medical curricula could enhance the learning experience, making it more immersive and interactive. This can be particularly beneficial in settings where TSM are limited or where there is a need for repeated practise without logistical constraints. This versatility would allow for a greater opportunity for simulation training for more students.

A systematic review by Tang et al. highlights challenges in validating small-scale AR studies but notes consistently positive feedback from participants and facilitators, a sentiment echoed in this study regarding AR's novelty [17]. AR is widely applied in medical education, but larger, long-term studies are needed for stronger evidence [17]. While this study offers insights into AR's role in WRBS, randomised controlled trials comparing it to standard ward-based teaching would be more conclusive. Future studies should address potential response bias to further validate these findings and explore the long-term retention of confidence gained through these simulations. Despite limited robust evidence for AR in curricula, educators are constantly adapting to engage their students in novel ways. This may call for innovative solutions to facilitate the delivery of an otherwise complex or unpopular topic [23].

Qualitative insights

The primary strength of AR identified by students was its visual accuracy, which allowed for independent recognition of clinical signs. Many students noted that the visual realism of AR surpassed that of manikin and TSM, enhancing their confidence and providing a unique educational experience. A systematic review of AR in interventional radiology highlights its benefits in enhancing realism, particularly for visuo-spatial awareness and procedural accuracy [24]. Additionally, Loeb et al. discuss fidelity and realism in AR, reporting that it offers visual and cognitive fidelity, resulting in more reliable physical examination findings [25]. They emphasise that AR can emulate clinical environments and real-life experiences, though they suggest this benefit is more advantageous for advanced learners. Furthermore, they argue that the illusion of realism is diminished if participants cannot physically interact with the digital representation, while TSM excel in teaching practical and communication skills to novices. This immersive tool addresses potential gaps in clinical exposure to acutely unwell patients, allowing students to demonstrate important clinical signs in a safe environment. The fear of making mistakes during real ward rounds also highlights the need for supportive training settings. Notably, Toohey et al. demonstrated in their study that AR simulations did not induce higher stress than manikin-based simulations, further supporting AR as an appropriate training modality that can maintain psychological safety while providing realism [26].

However, the lack of interactivity with the AR patient emerged as a substantial disadvantage. Students were unable to communicate with or physically examine the AR patient, which reduced the overall learning experience. The AR patient on its own limits the opportunity for dynamic learning and the development of communication skills. This lack of interaction also diminished the emotional engagement of students, leaving a gap in the acquisition of important communication skills. Although the AR modality utilised in this study lacked an interactivity component, other studies have demonstrated that incorporating interactive tools can enhance both theoretical knowledge and practical skills through AR [16]. While the application of AR is more established in surgical skill training, these same principles can be adapted to improve communication and procedural examination skills in medical consultation settings. This suggests a potential for improvement in future adaptations of AR.

The novelty of AR was both a strength and a challenge. While it excited and engaged students, the unfamiliarity with the technology added to the cognitive load. The mixed-methods approach, while beneficial for exposing students to various aspects of patient care, can itself be seen as a stressor. Cognitive load theory suggests that unfamiliar elements can strain working memory, potentially hindering the learner's ability to process new information effectively [27]. Switching between different modalities requires continuous adaptation; this may impede the ability to process the simulation content and learn effectively. Prior training and exposure to AR simulation will help overcome this barrier and reduce cognitive burden, allowing for greater focus on the clinical ward round experience and experiential learning. Despite its novelty, the literature suggests that repeated digitalised simulations can lead to a plateau in learning [24]; this calls for a diversified and embedded approach as incorporated into this WRBS.

From a faculty perspective, there were difficulties in ensuring that the technology was correctly set up and consistently working prior to each group. Although this was not noticed by students or discussed in the focus group, the technology was not always reliably operational in preparation for the students adding to additional stress and time needed when setting up each session. Despite this, the mixed-method WRBS required less faculty than previous attempts. The simulation can be run in any large open plan room with good Wi-Fi connectivity, reducing the need for a highly sought-after and expensive high-fidelity simulation suite. This study has an impact on resource efficiency; using this session plan, a WRBS simulation can be done in a more accessible location with a minimum faculty of three. However, it does require the acquisition of AR headsets, familiarity with the technology, and reliable internet connectivity. Dhar et al. note that a big hurdle in the use of AR is making the technology equitable, affordable, and accessible on a large scale [16]. It also highlights the potential dangers of AR contributing to social isolation associated with digitalised learning [16]. However, this study uses an embedded approach necessitating the need for teamwork and tutor facilitation.

## Conclusions

Both the quantitative and qualitative findings underscore the potential benefits of incorporating AR into medical training. While this is a mixed-methods study, the strengths lie in the qualitative data analysis, which highlights AR's role in improving visual fidelity. However, addressing the limitations, such as the lack of interactivity and the initial cognitive load, is crucial for optimising its effectiveness. The significant improvements in confidence and the enhanced realism of AR suggest that it can be a valuable tool in medical education. The quantitative data, although useful, is not conclusive in directly comparing AR with TSM. Future studies should focus on these areas, exploring how AR can be integrated with other modalities and how prior exposure can reduce cognitive burden. The study’s integrated insights highlight the need for continued innovation and adaptation in medical simulation technologies. By leveraging the strengths of both quantitative and qualitative data, medical educators can better understand the impacts of different simulation modalities and develop more effective, immersive, and interactive training programs.

## Appendices

Appendix 1

**Table 4:** Details of simulated patient cases used in the WRBS and their progression plan. AR: augmented reality; WRBS: ward round-based simulation; CAP: community-acquired pneumonia; AMTS: abbreviated mental test score; COPD: chronic obstructive pulmonary disease; NOK: next of kin; PPI: proton pump inhibitor; AKI: acute kidney injury

Patient	Clinical case	Ward Round Plan	During sim
1. Actor: 82-year-old female patient	Day 2 of admission. Severe CAP-on IV antibiotics. Fell on the ward yesterday with a head injury. Nursing staff concerned about increased confusion	Confusion screen, AMTS, collateral history, needs urgent CT head	CT scan request, nurse to ask if normal meds to be given, increasingly confused/disorientated. If sim goes well, can consider prompting for collateral history
2. AR: 68-year-old male patient	Severe CAP-on IV vancomycin and clarithromycin. Known diabetes, hypertension, COPD. Deteriorates prior to ward round	Sepsis 6, update NOK, ceiling of treatment	Nurse points out penicillin allergy. Add PPI cover
3. Manikin: 91-year-old male patient	Day 2 of admission. Known multiple myeloma. Increased confusion for three weeks, not coping at home. Worsening renal failure	Chase haematology and renal referral, collateral history, chase re-feeding blood, monitor U/O (chase USS report if patient off ward)	Hypercalcaemia 3.2, management of worsening AKI (drug chart review and fluids)

Appendix 2 

**Table 5:** Questions posed to the medical student focus groups.

Questions asked in the focus group	
1	How did you find the augmented reality?
2	How did augmented reality affect your learning?
3	How did the traditional methods, the actor, and the manikin affect your learning today?

Appendix 3 

**Table 6:** Questionnaire given to students to complete before and after the WRBS, referred to as pre- and post-questionnaires. WRBS: ward round-based simulation; SBAR: situation, background, assessment, and recommendation

Question number	Question
1	How confident do you feel actively participating in a ward round led by someone more senior? For instance, scribing. 1 = Not confident at all; 10 = Extremely confident
2	How confident do you feel in undertaking a ward round by yourself? 1 = Not confident at all; 10 = Extremely confident
3	How confident do you feel in your assessment and examination of the patient during a ward round? 1 = Not confident at all; 10 = Extremely confident
4	How confident do you feel coming up with the plan following your assessment of the patient during a ward round? 1 = Not confident at all; 10 = Extremely confident
5	How confident do you feel requesting investigations following a ward round? 1 = Not confident at all; 10 = Extremely confident
6	How confident do you feel about your SBAR handovers? For instance, when vetting a scan. 1 = Not confident at all; 10 = Extremely confident
7	How confident do you feel knowing when and how to escalate problems during the ward round? 1 = Not confident at all; 10 = Extremely confident
8	How confident do you feel stopping and/or starting medications during a ward round? 1 = Not confident at all; 10 = Extremely confident
9	How confident do you feel about creating and prioritising a job list following a ward round? 1 = Not confident at all; 10 = Extremely confident
10 (Pre-WRBS only)	Have you done any virtual reality or augmented reality simulations before? Please provide details if you have participated before.
11 (Post-WRBS only)	I found the augmented reality patient useful for my learning. Neutral = 0; Agree = 1; Strongly agree = 2
12 (Post-WRBS only)	I found the traditional simulation (actor + manikin) useful for my learning. Neutral = 0; Agree = 1; Strongly agree = 2
